# Prognostic significance of ypN status after neoadjuvant chemoimmunotherapy in resectable NSCLC: a systematic review and meta-analysis

**DOI:** 10.3389/fonc.2026.1842157

**Published:** 2026-05-22

**Authors:** Huan Shao, Lingyun Zou, Xiaojiao Zhu, Yingding Ruan, Hongsheng Xue

**Affiliations:** 1 Department of Oncology, The First People’s Hospital of Jiande, Jiande, China; 2 Department of Thoracic Surgery, Affiliated Zhongshan Hospital of Dalian University, Dalian, China; 3 Department of Thoracic Surgery, The First People’s Hospital of Jiande, Jiande, China

**Keywords:** meta-analysis, neoadjuvant chemoimmunotherapy, non-small cell lung cancer, prognosis, ypN status

## Abstract

**Background:**

Neoadjuvant chemoimmunotherapy has become an important treatment strategy for resectable non-small cell lung cancer (NSCLC), yet postoperative relapse remains common. Although pathological response is increasingly used for prognostication, the prognostic value of post-treatment nodal status remains unclear. We therefore conducted a systematic review and meta-analysis to evaluate the association between ypN status and survival outcomes after neoadjuvant chemoimmunotherapy in resectable NSCLC.

**Methods:**

In accordance with PRISMA 2020 guidelines, PubMed, Web of Science, Scopus, Embase, and the Cochrane Library were searched from inception to February 26, 2026. Studies reporting survival outcomes stratified by pathological nodal response in patients with resectable NSCLC treated with neoadjuvant chemoimmunotherapy were included. Overall survival (OS) was prespecified as the primary outcome. Disease-free survival (DFS) was considered a secondary outcome, while recurrence-free survival (RFS) was summarized descriptively when quantitative pooling was not feasible. Hazard ratios (HRs) with 95% confidence intervals (CIs) were pooled using fixed- or random-effects models according to heterogeneity.

**Results:**

Two studies were included in the primary meta-analysis of OS. Across prespecified clinically comparable but non-identical nodal-response contrasts, unfavorable post-treatment nodal status was associated with significantly poorer OS (pooled HR 4.75, 95% CI 2.38 - 9.47; P < 0.00001; I² = 0%). A similar association was observed for DFS (pooled HR 3.54, 95% CI 1.71–7.33; P = 0.0007; I² = 0%). Sensitivity analyses showed consistent results. For RFS, only one study provided extractable data, suggesting that combined major pathological response (MPR)-ypN classification may further stratify prognosis, with the non-MPR ypN+ group showing the poorest outcome.

**Conclusions:**

Across prespecified clinically comparable but non-identical nodal-response contrasts, unfavorable post-treatment nodal status was associated with poorer survival outcomes in resectable NSCLC. Because the pooled OS and DFS analyses each included only two retrospective studies, these findings should be interpreted cautiously pending prospective validation with standardized pathological classifications and uniform survival endpoints.

**Systematic review registration:**

https://www.crd.york.ac.uk/prospero/, identifier CRD420261353461.

## Introduction

The treatment landscape of resectable non-small cell lung cancer (NSCLC) has changed substantially with the introduction of neoadjuvant and perioperative immunotherapy-based strategies. In CheckMate 816, adding nivolumab to platinum-doublet chemotherapy significantly increased pathological complete response and prolonged event-free survival compared with chemotherapy alone (1). Subsequent phase III trials further extended this approach into the perioperative setting. In KEYNOTE-671, perioperative pembrolizumab plus neoadjuvant chemotherapy improved event-free survival and major pathological response, and later analyses also showed an overall survival benefit (2, 3). Similarly, AEGEAN demonstrated that perioperative durvalumab plus neoadjuvant chemotherapy improved pathological complete response and event-free survival in resectable NSCLC (4). A recent systematic review and meta-analysis also confirmed that, compared with neoadjuvant chemotherapy alone, neoadjuvant chemoimmunotherapy is associated with superior pathologica and efficacy outcomes across randomized studies (5).

Despite these advances, postoperative recurrence remains a major challenge, and not all patients derive the same long-term benefit after surgery. One reason is that the major registration trials adopted different treatment paradigms: CheckMate 816 evaluated a purely neoadjuvant strategy, whereas KEYNOTE-671 and AEGEAN incorporated adjuvant immunotherapy after resection (1–4). This difference is clinically important, because it means that postoperative treatment intensity cannot be determined solely from trial success in the overall population. In routine practice, the key question is no longer whether neoadjuvant chemoimmunotherapy works, but which postoperative pathological features best identify patients at persistently high risk of recurrence and therefore most likely to benefit from closer surveillance or treatment intensification.

Post-neoadjuvant pathological assessment is the most practical basis for such risk stratification. The IASLC has provided standardized recommendations for pathological evaluation of resection specimens after neoadjuvant therapy, including the assessment of complete pathological response and major pathological response (MPR) (6). These endpoints are clinically attractive because they are available immediately after surgery and can be incorporated into postoperative decision-making. However, recent analyses have also shown that the relationship between pathological response and long-term survival is more nuanced than initially assumed. In a trial-level meta-analysis of randomized neoadjuvant immunotherapy studies, both pCR and MPR correlated strongly with 2-year event-free survival, but their surrogacy for overall survival was more limited and imprecise (7). Likewise, a large pooled pathological-response analysis suggested that residual viable tumor thresholds beyond conventional pCR and MPR may further refine prognosis, highlighting that pathologic response is not fully captured by a single binary cutoff (8). Together, these data suggest that pathological regression in the primary tumor is informative, but may not be sufficient on its own to define postoperative risk.

Within this context, pathological nodal status after neoadjuvant chemoimmunotherapy has attracted increasing attention. Residual nodal disease, even in the presence of primary tumor regression, may reflect incomplete systemic control and could provide prognostic information beyond MPR alone. Emerging retrospective studies support this concept, but the available evidence remains limited and heterogeneous. Some studies have focused on simple ypN0 versus ypN+ comparisons, whereas others have further distinguished natural node-negative from downstaged node-negative disease or incorporated lymph node pathological response and combined MPR-ypN classification models, suggesting that integration of nodal status and primary tumor response may improve prognostic stratification compared with either alone (9–11). However, recent studies published between 2023 and 2026 have also differed substantially in sample size, subgroup definitions, follow-up duration, and reported survival endpoints, including overall survival, disease-free survival, recurrence-free survival, and progression-free survival (9–13). Such variability in both classification strategies and outcome definitions limits cross-study comparability and complicates interpretation of the prognostic role of ypN status after neoadjuvant chemoimmunotherapy.

Given these uncertainties, we conducted a systematic review and meta-analysis to evaluate the prognostic significance of post-treatment nodal status in patients with resectable NSCLC treated with neoadjuvant chemoimmunotherapy followed by surgery. Our aim was to determine whether unfavorable post-treatment nodal status, across clinically comparable but heterogeneous nodal-response definitions, is consistently associated with poorer survival outcomes and to clarify the role of postoperative nodal assessment in risk stratification within this evolving treatment landscape.

## Methods

### Protocol and reporting standard

This systematic review and meta-analysis was conducted in accordance with the Preferred Reporting Items for Systematic Reviews and Meta-Analyses (PRISMA) 2020 statement (14) and the methodological recommendations of the Cochrane Handbook for Systematic Reviews of Interventions (15). The completed PRISMA 2020 checklist, including page and line references for each item, is provided as **Supplementary Material S1**. The review protocol was prospectively registered in the International Prospective Register of Systematic Reviews (PROSPERO; registration No. CRD420261353461).

### Search strategy

A comprehensive literature search was independently performed by two reviewers in PubMed, Embase, Web of Science Core Collection, Scopus, and the Cochrane Library from database inception to 26 February 2026. The search strategy combined controlled vocabulary and free-text terms across four core domains: non-small cell lung cancer, neoadjuvant chemoimmunotherapy, pathological nodal status, and survival outcomes. Terms within each domain were combined with OR, and the domains were then combined with AND. The strategy was adapted to the indexing system and syntax of each database. In addition, the reference lists of all eligible studies and relevant review articles were manually screened to identify any additional studies. The detailed search strategies for all databases are provided in the **Supplementary Material**.

### Eligibility criteria

Studies were included if they met the following criteria: (1) patients had histologically confirmed resectable or potentially resectable NSCLC; (2) patients received neoadjuvant chemoimmunotherapy followed by surgical resection; (3) postoperative pathological nodal status (ypN) was reported; and (4) at least one survival outcome of interest, including overall survival (OS), disease-free survival (DFS), or recurrence-free survival (RFS), was reported according to post-treatment nodal status or in a form allowing extraction or estimation of comparative effect sizes. The primary outcome was OS. DFS was prespecified as a secondary outcome, and RFS was summarized descriptively when quantitative pooling was not feasible.

Studies were excluded if they: (1) included patients treated with neoadjuvant chemotherapy alone or immunotherapy alone without separable chemoimmunotherapy data; (2) included unresectable disease, metastatic disease, non-NSCLC histologies, or mixed populations without extractable data for resectable NSCLC; (3) did not report postoperative pathological nodal status or did not provide a nodal status–based prognostic comparison; (4) lacked extractable survival data; or (5) were reviews, editorials, letters, case reports, conference abstracts, protocols, or duplicate publications. When multiple reports involved overlapping cohorts, the most informative study with the most complete prognostic data or longest follow-up was retained.

### Study selection

Two reviewers independently screened all retrieved records in two stages. After removal of duplicates, titles and abstracts were screened for potential relevance. Full texts of potentially eligible studies were then reviewed against the predefined inclusion and exclusion criteria. Disagreements were resolved through discussion, with consultation of a third reviewer when necessary.

### Data extraction

Data were independently extracted by two reviewers using a standardized form. The following information was collected: first author, publication year, country, study design, study population, sample size, baseline clinicopathological characteristics, treatment details, pathological nodal response categories, pathological response variables, and reported survival outcomes. For time-to-event outcomes, hazard ratios (HRs) and corresponding 95% confidence intervals (CIs) were extracted preferentially from multivariable analyses when available. When adjusted estimates were unavailable, univariable HRs were extracted and used for descriptive or sensitivity analyses, but they were not prioritized for the primary pooled analysis.

To avoid double counting in the primary meta-analysis when multiple HRs were reported against a shared reference group within the same study, one prespecified eligible comparison from each study was retained for the main pooled analysis. This comparison was selected according to a hierarchical rule that prioritized: (1) post-treatment pathological nodal status–based comparisons over composite or exploratory subgroup models; (2) contrasts judged to be most clinically comparable across studies; and (3) estimates derived from the overall study population rather than restricted subgroups. Alternative eligible comparisons from the same source study were examined in sensitivity analyses. Clinically informative but non-independent subgroup structures, such as combined major pathological response (MPR)-ypN categories, were summarized descriptively when direct quantitative pooling was not appropriate.

### Quality assessment

The methodological quality of included non-randomized studies was independently assessed by two reviewers using the Newcastle–Ottawa Scale (NOS), which evaluates study quality across the domains of selection, comparability, and outcome, with scores ranging from 0 to 9. Discrepancies were resolved by discussion with a third reviewer when necessary.

### Statistical analysis

Meta-analyses were performed using Review Manager (RevMan) version 5.4.1. HRs with 95% CIs were pooled for time-to-event outcomes. For quantitative synthesis, HRs were transformed into log(HR) values and corresponding standard errors. Statistical heterogeneity was assessed using Cochran’s Q test and the I² statistic. An I² value <50% was considered low heterogeneity, and a fixed-effects model was used in such cases; otherwise, a random-effects model was applied. A two-sided P value < 0.05 was considered statistically significant.

The primary quantitative synthesis focused on OS according to post-treatment nodal status, as OS is the most definitive survival endpoint. DFS was prespecified as a secondary endpoint and analyzed separately when sufficiently comparable data were available. DFS, RFS, and PFS were treated as related but non-interchangeable recurrence-based endpoints because their event definitions, follow-up frameworks, and censoring rules may differ across studies; they were therefore not pooled together. RFS and PFS were summarized descriptively when quantitative synthesis was not appropriate because of single-study availability, inconsistent endpoint definitions, or shared-reference subgroup structures.

To preserve statistical independence, one prespecified comparison per study was retained when multiple nodal-response categories or HRs shared a common reference group. Because nodal-response definitions varied across studies, pooled estimates were interpreted as reflecting clinically comparable but non-identical exposure constructs rather than a single uniform definition of ypN response. Sensitivity analyses replaced the prespecified comparison with alternative eligible comparisons from the same study to assess robustness. Publication bias was not formally assessed because fewer than 10 studies were available for each pooled outcome.

## Results

### Study selection and characteristics

**Figure 1:**
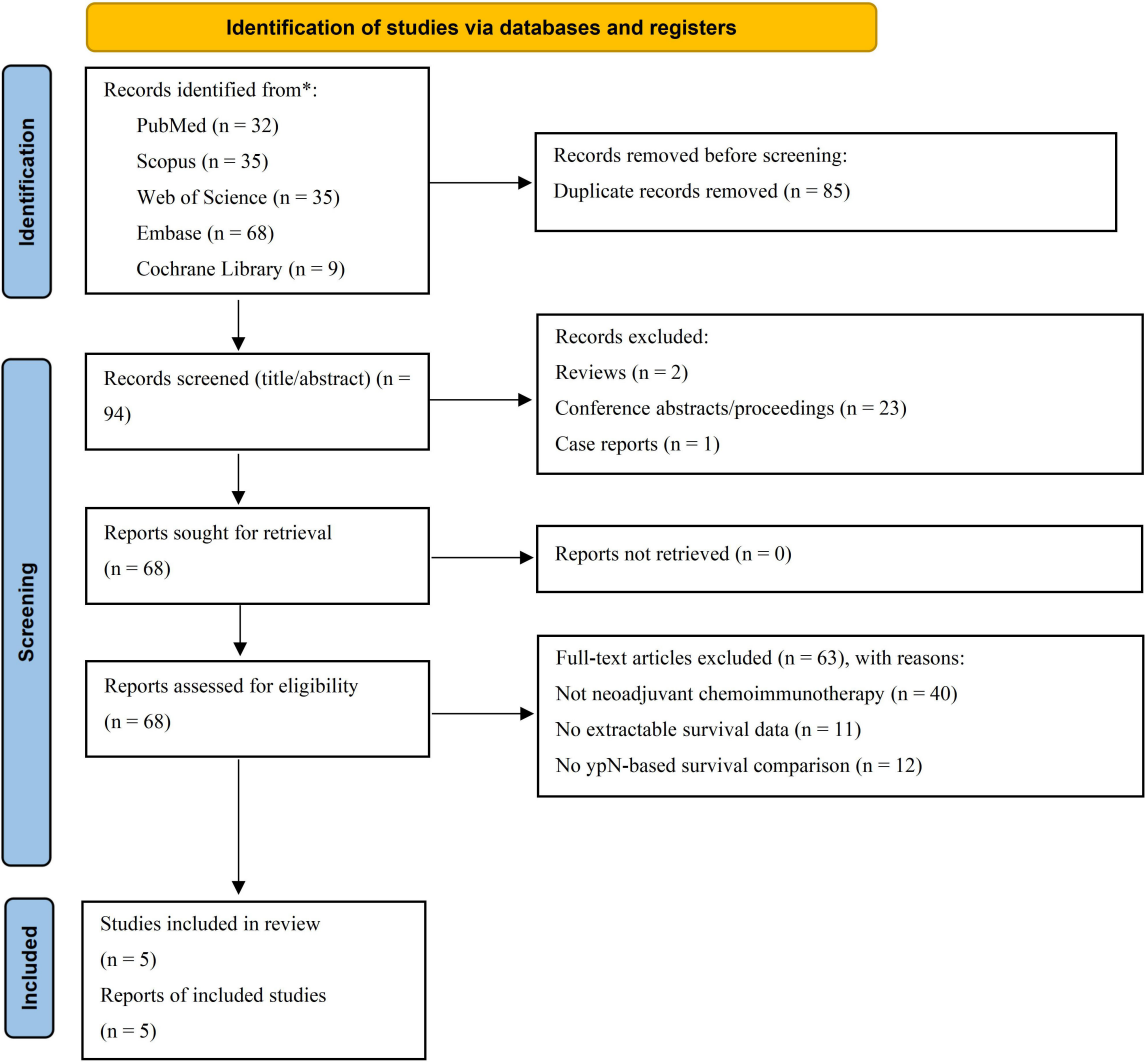
The PRISMA flow diagram displays the details of the selection process. *From: Page, M.J., McKenzie, J.E., Bossuyt, P.M., Boutron, I., Hofmann, T.C., Mulrow, C.D., Shamseer, L., Tetzlaf, J.M., Akl, E.A., Brennan, S.E., et al. (2021). The PRISMA 2020 statement: an updated guideline for reporting systematic reviews. Syst Rev. Mar 29;10(1):89. doi.org/10.1186/s13643-021-01626-4 PMID: 33781348; PMCID: PMC8008539.

From database inception to 26 February 2026, the systematic search identified a total of 179 records. After removal of 85 duplicates, 94 records were screened by title and abstract, and 68 articles underwent full-text assessment. Ultimately, five studies met the inclusion criteria and were included in the qualitative synthesis, all of which contributed data to at least one quantitative analysis. The number of studies contributing to each quantitative analysis differed according to outcome type, effect-estimate availability, and subgroup structure. Reasons for full-text exclusion are summarized in **Figure 1**.

All included studies were retrospective observational cohorts examining the prognostic relevance of pathological response and post-treatment nodal status following neoadjuvant chemoimmunotherapy in resectable non-small cell lung cancer (9–13). The studies were conducted in China or the United States and varied in sample size, baseline nodal status, treatment strategies, and pathological classification approaches. Although the definitions of major pathological response (MPR) and pathological nodal status (ypN) were broadly comparable, the specific grouping frameworks differed across studies, including simple ypN0 versus ypN+ comparisons (10), stratification into natural N0, downstaged N0, and ypN+ categories (11), lymph node MPR–based classification (9), and combined MPR–ypN subgroup models (11, 13).

Survival endpoints were reported inconsistently across studies. Overall survival (OS) was available in most cohorts (10–13), whereas recurrence-related outcomes were variably defined as disease-free survival (DFS) (9, 11), recurrence-free survival (RFS) (13), or progression-free survival (PFS) (12). Accordingly, the number of studies contributing to each meta-analysis differed by outcome. In some cases, multiple subgroup comparisons were reported within a single study, and not all were directly comparable across cohorts.

Key study characteristics are summarized in **Table 1**. Detailed extracted data are provided in **Supplementary Table 1**, and study quality assessment using the Newcastle–Ottawa Scale is presented in **Table 2**.

**Table 1:** Main characteristics of the included studies.

Characteristic	Deng et al. (9)	Du et al. (12)	Guo et al. (10)	Ma et al. (11)	Pan et al. (13)
Year; country	2023; China	2023; China	2025; United States	2025; China	2026; China
Study design	Retrospective cohort	Retrospective cohort	Retrospective database study	Retrospective multicenter cohort	Retrospective multicenter cohort
Population	Initial cStage III NSCLC treated with neoadjuvant immunochemotherapy and radical-intent surgery	Resectable NSCLC with baseline cN1 or cN2 nodal disease	Resectable NSCLC with baseline cN1 or cN2 disease treated with neoadjuvant chemoimmunotherapy and surgery	NSCLC after neoadjuvant chemoimmunotherapy and surgery	cStage IB–III resectable NSCLC treated with neoadjuvant chemoimmunotherapy followed by curative-intent surgery
Total sample size, n	53	75	621	186	363
ypN0 / nodal clearance, n	28	54	293	129	260
ypN+ / residual nodal disease, n	25	21	328	57	103
Nodal classification framework	ypN0 versus ypN1–N2; metastatic lymph node MPR also evaluated	Nodal clearance versus residual nodal disease	ypN0 versus ypN+; additional cN1/cN2 subgrouping	Natural node-negative, downstaged node-negative, and ypN+ groups	ypN0 versus ypN+; combined MPR–ypN classification
Treatment setting	Neoadjuvant immunochemotherapy + surgery	Neoadjuvant chemoimmunotherapy + surgery	Neoadjuvant chemoimmunotherapy + surgery	Neoadjuvant chemoimmunotherapy + surgery	Neoadjuvant chemoimmunotherapy + surgery; subgroup analysis of adjuvant IO
Main clinicopathological variables reported	Primary tumor response; metastatic lymph node response; residual viable tumor in lymph nodes; pathological nodal stage	Age; sex; smoking history; histology; baseline nodal burden; MPR; pCR; nodal clearance	cN stage; ypT stage; ypN stage; regional LNs examined; margin status; adjuvant chemoimmunotherapy; adjuvant radiation	Pretreatment LN status; cT stage; cN stage; ypT stage; ypN status; MPR; recurrence pattern	MPR; ypN status; cT stage; cN stage; ypT stage; recurrence pattern; adjuvant IO exposure
Survival outcomes reported	DFS	PFS; OS	OS	DFS; OS	RFS; OS

NSCLC, non-small cell lung cancer; cStage, clinical stage; cN, clinical nodal stage; cT, clinical T stage; ypT, post-treatment pathological T stage; ypN, post-treatment pathological nodal stage; ypN0, post-treatment pathological node-negative status; ypN+, post-treatment pathological node-positive status; MPR, major pathological response; pCR, pathological complete response; LN, lymph node; LNs, lymph nodes; DFS, disease-free survival; PFS, progression-free survival; OS, overall survival; RFS, recurrence-free survival; IO, immunotherapy.

**Table 2:** The Newcastle-Ottawa Scale (NOS) for assessing the quality of nonrandomized studies in our study.

Study	Year	Country	Type of article	The Newcastle-Ottawa Scale (NOS)		
				Selection	Comparability	Outcome
Deng et al. (9)	2023	China	Retrospective cohort	* * * *	–	*
Du et al. (12)	2023	China	Retrospective cohort	* * * *	–	* *
Guo et al. (10)	2025	United States	Retrospective database study	* * * *	* *	* * *
Ma et al. (11)	2025	China	Retrospective multicenter cohort	* * * *	* *	* * *
Pan et al. (13)	2026	China	Retrospective multicenter cohort	* * * *	* *	* * *

* = 1 point; maximum score = 9 points.

NOS was applied using the cohort-study domains: Selection, Comparability, and Outcome.

One star in the Comparability domain indicates adjustment for the most important prognostic confounder or confounders; two stars indicate additional adjustment for other relevant clinicopathological confounders.

"-" indicates that no star was awarded in that domain.

Across the included studies, the main sources of bias were related to their retrospective design and the variability in adjustment for confounding factors. Although most studies achieved high scores in the selection domain, comparability was limited in some cohorts because multivariable adjustment was incomplete or not consistently reported. In addition, differences in postoperative treatment strategies and follow-up protocols may have introduced further heterogeneity in outcome assessment. These factors should be considered when interpreting the pooled estimates.

### Primary outcomes

#### Overall survival

**Figure 2:**
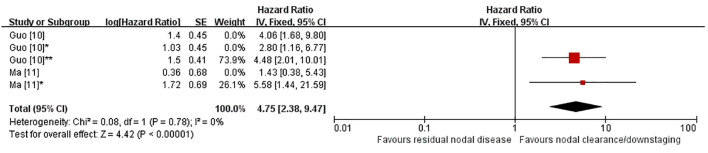
Primary meta-analysis of overall survival (OS) according to post-neoadjuvant nodal status, presented as a fixed-effect forest plot. To avoid double counting when multiple hazard ratios were reported against a shared reference group, one prespecified comparison from each study was retained in the primary analysis. OS, overall survival; HR, hazard ratio; CI, confidence interval. Guo (10) = cN1→ypN+ versus cN1→ypN−; Guo (10)* = cN2→ypN− versus cN1→ypN−; Guo (10)** = cN2→ypN+ versus cN1→ypN−; Ma (11) = downstaged N0 versus natural N0; Ma (11)* = ypN+ versus natural N0.

Two studies were included in the primary meta-analysis of overall survival (OS) (10, 11). Because both studies reported multiple hazard ratios against a shared reference group, one prespecified comparison from each study was selected for the primary analysis. Across two clinically comparable but non-identical nodal-response comparisons, unfavorable post-treatment nodal status was associated with worse OS than favorable nodal status (HR 4.75, 95% CI 2.38–9.47; P < 0.00001; **Figure 2**), with no heterogeneity detected (I² = 0%). The prespecified comparisons were cN2→ypN+ versus cN1→ypN− and ypN+ versus natural N0. Although the exposure definitions were not identical, both comparisons suggested that residual nodal disease or an unfavorable post-treatment nodal-response pattern was associated with inferior long-term survival (10, 11).

Sensitivity analyses were performed by substituting alternative eligible comparisons. Using cN1→ypN+ versus cN1→ypN− in place of the prespecified comparison yielded a similar result (HR 4.46, 95% CI 2.13–9.34; P < 0.0001; **Supplementary Figure 1**), again without heterogeneity (I² = 0%). Substitution with cN2→ypN− versus cN1→ypN− also retained statistical significance (HR 3.44, 95% CI 1.64–7.20; P = 0.001; **Supplementary Figure 2**), with I² remaining 0% (10). In contrast, replacing ypN+ versus natural N0 with downstaged N0 versus natural N0 attenuated the pooled estimate and rendered it non-significant under the random-effects model (HR 2.88, 95% CI 0.97–8.56; P = 0.06; **Supplementary Figure 3**), with moderate heterogeneity (I² = 51%) (11). Despite variation in effect size, the direction of association remained consistent across sensitivity analyses.

An exploratory analysis including all five eligible comparisons also showed poorer OS in patients with unfavorable nodal status (HR 3.53, 95% CI 2.28–5.46; P < 0.00001; **Supplementary Figure 4**), with I² = 0%. This result remains exploratory because several comparisons were drawn from the same studies and were not independent (10, 11).

### Secondary outcomes

#### Disease-free survival

**Figure 3:**
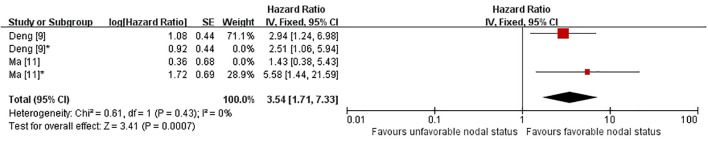
Primary meta-analysis of disease-free survival (DFS) according to post-neoadjuvant nodal status, presented as a fixed-effect forest plot. To avoid double counting when multiple hazard ratios were reported within the same study, one prespecified comparison from each study was retained in the primary analysis. DFS, disease-free survival; HR, hazard ratio; CI, confidence interval. Deng (9) = mLN-MPR(-) versus mLN-MPR(+); Deng (9)* = ypN1-N2 versus ypN0; Ma (11) = downstaged N0 versus natural N0; Ma (11)* = ypN+ versus natural N0.

Two studies were included in the primary meta-analysis of disease-free survival (DFS) (9, 11). Because both studies provided more than one eligible hazard ratio, one prespecified comparison from each study was selected for the primary analysis. Across heterogeneous but clinically related nodal-response frameworks, unfavorable nodal response was associated with poorer DFS than favorable nodal response (HR 3.54, 95% CI 1.71–7.33; P = 0.0007; **Figure 3**), with no heterogeneity detected (I² = 0%). The prespecified comparisons were mLN-MPR(-) versus mLN-MPR(+) and ypN+ versus natural N0. These findings should be interpreted as reflecting a consistent adverse prognostic signal across related, but not identical, nodal-response constructs (9, 11).

Sensitivity analyses were performed by substituting alternative eligible comparisons. Using ypN1-N2 versus ypN0 in place of the prespecified comparison yielded a similar result (HR 3.16, 95% CI 1.53–6.54; P = 0.002; **Supplementary Figure 5**), again without heterogeneity (I² = 0%) (9). Replacing ypN+ versus natural N0 with downstaged N0 versus natural N0 attenuated the pooled estimate, although the association remained statistically significant (HR 2.38, 95% CI 1.15–4.91; P = 0.02; **Supplementary Figure 6**), with I² remaining 0% (11). Despite some variation in effect size, the direction of association remained consistent across sensitivity analyses.

An exploratory analysis including all four eligible comparisons also showed poorer DFS in patients with unfavorable nodal status (HR 2.74, 95% CI 1.64–4.58; P = 0.0001; **Supplementary Figure 7**), with I² = 0%. This result remains exploratory because several comparisons were drawn from the same studies and were not independent (9, 11).

#### Recurrence-free survival

Only one study reported extractable recurrence-free survival (RFS) data (13). In that study, ypN0 status and achievement of major pathological response (MPR) were both associated with better RFS, and the combined MPR-ypN classification further stratified prognosis. Using MPR ypN0 as the reference group, multivariable analysis showed worse RFS for non-MPR ypN0 (HR 2.21, 95% CI 1.07–4.56), MPR ypN+ (HR 5.54, 95% CI 2.66–11.54), and non-MPR ypN+ (HR 5.18, 95% CI 2.80–9.55). The non-MPR ypN+ group had the poorest outcome, with a 2-year RFS rate of 55.2% (13). Quantitative pooling was not performed because all reported comparisons came from a single study and shared a common reference group. For visual presentation, the three multivariable RFS comparisons were displayed in a single-study forest plot (**Supplementary Figure 8**); no pooled summary estimate, pooled diamond, heterogeneity statistic, or overall-effect test was calculated or displayed.

## Discussion

In this meta-analysis of a limited number of retrospective studies, unfavorable post-treatment nodal status or poor nodal response was associated with worse survival after neoadjuvant chemoimmunotherapy in resectable NSCLC. However, the evidence remains limited, and the pooled exposure definitions were clinically comparable rather than identical. These findings support further evaluation of ypN status as a postoperative prognostic marker, particularly in combination with primary-tumor response.

From a clinical standpoint, the prognostic value of ypN status after neoadjuvant chemoimmunotherapy is both biologically plausible and clinically consequential. Although major pathologic response (MPR) and pathologic complete response (pCR) in the primary tumor serve as established efficacy markers, they do not fully capture the systemic disease burden. Our findings reinforce that nodal assessment provides nonredundant prognostic stratification: even in cases of marked primary tumor regression, persistent nodal disease likely signifies a higher burden of residual micrometastatic spread. This is corroborated by the individual cohorts included in our analysis. Ma et al. demonstrated that ypN+ patients had significantly worse DFS and OS than both natural N0 and downstaged N0 groups, whereas outcomes were comparable between the latter two, suggesting that residual nodal disease—rather than baseline nodal status—is the principal driver of postoperative risk (11). Similarly, Guo et al. reported that post-therapy nodal stage carried clearer long-term prognostic value than baseline clinical stage, supporting ypN status as a clinically relevant post-treatment stratifier (10).

Biologically, persistent nodal disease may represent more than residual locoregional disease; it may indicate incomplete systemic response and a continuing risk of relapse. Tumor-draining lymph nodes function not only as anatomic conduits for metastasis but also as critical immune-regulatory hubs governing antitumor T-cell priming and tolerance (16–18). As nodal metastases evolve, this microenvironment may shift toward immunosuppression, fostering the persistence of treatment-resistant clones and further dissemination (17, 19). In this context, ypN+ status may capture a component of residual risk that is not fully represented by primary tumor regression alone. This biological context may help explain why combined models outperform single metrics. Pan et al. showed that integrating MPR with ypN status further refined prognostic separation, with the non-MPR/ypN+ subgroup exhibiting the poorest recurrence-free survival (13). Taken together, these observations support the hypothesis that ypN status may provide complementary prognostic information to primary-tumor response, although this interpretation remains inferential and requires prospective validation.

The OS findings are clinically informative because the adverse association between unfavorable post-treatment nodal status and survival remained directionally consistent across the primary and sensitivity analyses, despite differences in subgroup construction. Whether unfavorable nodal status was defined as residual ypN+ disease, cN-to-ypN transition patterns, or subgroup models incorporating pretreatment nodal burden, the association generally pointed toward inferior long-term survival. However, these comparisons should not be interpreted as representing a single homogeneous exposure definition. This pattern suggests that the prognostic signal of residual nodal disease may not be limited to one comparison framework.

This observation is relevant in the context of recent perioperative immunotherapy studies, in which improvements in pathological response and event-free endpoints have often matured earlier than overall survival and have not always translated into OS with the same precision. In a trial-level analysis, Hines et al. showed that pCR and MPR correlated strongly with 2-year EFS, whereas their correlations with 2-year OS were weaker, suggesting that primary-tumor regression alone may not fully account for long-term survival risk (7). The updated survival analysis of CheckMate 816 further illustrates the importance of OS in this setting: with a median follow-up of 68.4 months, neoadjuvant nivolumab plus chemotherapy achieved a 5-year OS of 65.4%, compared with 55.0% for chemotherapy alone (20). Similarly, perioperative pembrolizumab in KEYNOTE-671 improved 36-month OS from 64% to 71% (HR 0.72, 95% CI 0.56–0.93) (3). Recent methodological discussions have emphasized that early surrogate endpoints in the perioperative immunotherapy era should be interpreted cautiously (21, 22). A contemporary meta-analysis also reported a significant OS benefit with neoadjuvant or perioperative immunotherapy strategies, while noting variability across intermediate endpoints and treatment paradigms (23). Against this background, our pooled analysis suggests that ypN status may capture a component of postoperative risk not fully reflected by primary-tumor regression alone. These observations support further evaluation, rather than immediate clinical adoption, of post-treatment nodal status as part of postoperative risk stratification after neoadjuvant chemoimmunotherapy.

The DFS findings were directionally consistent with the OS results, but should be interpreted as supportive rather than definitive because the underlying nodal-response frameworks differed across studies. In the primary analysis, unfavorable post-treatment nodal status was associated with significantly worse DFS (HR 3.54, 95% CI 1.71–7.33), and the same direction was maintained in sensitivity analyses using alternative comparison strategies, with HRs of 3.16 and 2.38. The main difference from the OS analysis lay in how nodal response was defined. Deng et al. used a modified lymph node major pathological response framework, whereas Ma et al. applied conventional ypN-based subgrouping and further distinguished natural node-negative from downstaged node-negative disease (9, 11). Although these approaches are not identical and should not be treated as interchangeable definitions of ypN response, both linked poor nodal response to a higher risk of recurrence.

This is also in keeping with the broader perioperative literature, where recurrence-based endpoints tend to be more sensitive than OS to differences in endpoint definition and subgroup construction (7, 22). In AEGEAN and CheckMate 77T, perioperative immunotherapy was associated with significant event-free survival benefit (4, 24). In the exploratory stage III analysis of CheckMate 77T, perioperative nivolumab improved 1-year EFS from 45% to 70% in patients with N2 disease (HR 0.46, 95% CI 0.30–0.70) (25). These findings suggest that pathological response, nodal downstaging, and residual nodal disease reflect related but not identical aspects of postoperative risk. The DFS result in the present study should therefore be viewed as supportive rather than definitive, and further progress in this area will depend on more standardized nodal-response definitions and more consistent reporting of recurrence-related endpoints.

The RFS findings are particularly noteworthy because they suggest that postoperative risk may not be fully captured by any single pathological marker. Only one study provided extractable RFS data, so quantitative pooling was not appropriate. The pattern reported by Pan et al., however, is striking. Using MPR/ypN0 as the reference, non-MPR/ypN0 was associated with worse RFS (HR 2.21, 95% CI 1.07–4.56), and the risk increased further when residual nodal disease was present, both in the MPR/ypN+ group (HR 5.54, 95% CI 2.66–11.54) and the non-MPR/ypN+ group (HR 5.18, 95% CI 2.80–9.55). The poorest 2-year RFS was observed in the non-MPR/ypN+ subgroup, at 55.2% (13). This pattern suggests that MPR and ypN are not redundant readouts. Primary-tumor response alone does not eliminate recurrence risk, and nodal clearance alone does not fully define prognosis.

Similar observations have been reported in studies that examined pathological response and nodal status together rather than in isolation. In a real-world analysis of 204 patients, Shao et al. found that a combined ypTMPR/ypN framework separated progression-free survival more clearly than either parameter alone: the ypTMPR(+)/ypN0 group had the best prognosis, whereas ypTMPR(-)/ypN1–2 had the worst, with mean PFS times of 35.7 and 28.7 months, respectively (26). Li et al. likewise showed that these pathological dimensions were not interchangeable: residual viable tumor in the primary lesion was independently associated with RFS (HR 3.96, 95% CI 1.30–12.01), while mediastinal lymph node recurrence increased from 3.8% in ypN0 disease to 15.0% in ypN1 and 25.0% in ypN2 (27). Data from Fudan further suggest that postoperative benefit from adjuvant ICI may be concentrated in patients without pCR or MPR, implying that primary-tumor response continues to modify residual postoperative risk (28). Even pCR does not abolish recurrence completely; in a 130-patient series, 11 recurrences were observed after pCR, and 10 occurred within 18 months (29). These observations support a more integrated view of postoperative pathology. ypN may identify residual risk even in patients who appear to have responded well in the primary tumor, whereas primary-tumor response may still refine recurrence risk among patients who achieve nodal clearance. For that reason, the RFS findings in this study are best understood as an early signal that postoperative risk stratification after neoadjuvant chemoimmunotherapy is likely to require joint interpretation of primary-tumor response and nodal status rather than reliance on either alone.

With the rapid adoption of neoadjuvant and perioperative immunotherapy in resectable NSCLC, the central clinical question has begun to shift. Trials such as CheckMate 816, KEYNOTE-671, and AEGEAN have established that immunotherapy-based perioperative strategies can improve pathological response and event-related outcomes; in CheckMate 816, for example, pCR increased from 2.2% to 24.0% with nivolumab plus chemotherapy, and the later survival analysis showed a 5-year OS of 65.4% versus 55.0% (1, 20). In KEYNOTE-671, the 36-month OS rate was 71% with perioperative pembrolizumab versus 64% with placebo (3). These studies answered the question of whether perioperative immunotherapy can improve outcomes. What they do not fully resolve, however, is how postoperative risk should be interpreted once resection has been completed. In routine practice, the issue is no longer simply whether chemoimmunotherapy works, but which pathological features still identify patients who remain at high risk despite apparently successful multimodality treatment.

The present findings may have clinical relevance, but they should not be interpreted as establishing ypN status as a stand-alone decision rule. ypN status is practical because it is available immediately after surgery and embedded in routine pathological assessment; however, its prognostic role remains insufficiently validated because the available evidence is limited to a small number of retrospective studies. It is best viewed as a candidate component of a broader postoperative risk profile that includes primary-tumor response, pretreatment stage, and perioperative treatment context (6, 30–33). Residual nodal disease may help identify patients who warrant closer follow-up, but this possibility should be tested in future studies evaluating risk-adapted postoperative strategies.

This study has several strengths. It addresses a focused and clinically relevant question in an area where the available evidence remains limited and methodologically heterogeneous. The review was prospectively registered and conducted in accordance with PRISMA 2020, which helped ensure a transparent and systematic process for study identification, selection, data extraction, and quality assessment. A particular strength of this meta-analysis is the prespecified handling of non-independent subgroup comparisons. By prioritizing statistical independence in the primary analyses and using sensitivity analyses to examine alternative eligible contrasts, we reduced the risk of overstating the precision of pooled estimates. We also distinguished between outcomes that could be synthesized quantitatively and findings that were more appropriately summarized descriptively, allowing the evidence to be presented without forcing clinically relevant but methodologically non-independent data into a pooled framework.

Several limitations should also be acknowledged. The evidence base was small: only five retrospective cohort studies met the eligibility criteria, and only two studies contributed to each primary pooled survival analysis. This substantially limited statistical power, reduced the precision and reliability of the pooled hazard ratios, and restricted the generalizability of the findings. The small number of studies also limited assessment of between-study heterogeneity and made any formal evaluation of publication bias uninformative. The included studies were also heterogeneous in several important respects, including nodal-response classification, subgroup structure, baseline nodal stage, perioperative treatment strategy, and outcome reporting. Some cohorts used simple ypN0 versus ypN+ comparisons, whereas others applied more refined frameworks such as natural N0, downstaged N0, lymph node MPR, or combined MPR-ypN subgrouping. Natural N0 and downstaged N0 patients are both ypN0 after surgery, but they differ in pretreatment nodal status and may not share the same baseline risk. Therefore, the pooled estimates should be interpreted as summarizing a shared adverse nodal-response signal across clinically comparable constructs, rather than as estimates for one uniform exposure or reference-group definition. These differences reduced direct comparability across studies and required careful prespecification of which contrasts could reasonably be pooled. Although the Newcastle–Ottawa Scale was used to assess study quality, it does not provide a formal certainty-of-evidence rating. It also may not fully capture all sources of bias in retrospective observational studies. Because all included studies were retrospective cohorts, the overall certainty of evidence should be considered low. The pooled estimates therefore remain vulnerable to residual confounding, selection bias, and heterogeneity in postoperative treatment, surveillance intensity, and follow-up duration. Accordingly, ypN status should be regarded as a promising rather than validated postoperative prognostic factor at this stage. Prospective multicenter studies using standardized nodal-response classifications and uniform survival endpoints are needed to clarify how ypN status should be integrated with primary-tumor response and other postoperative risk markers.

Post-treatment nodal status may capture a clinically relevant component of residual risk after neoadjuvant chemoimmunotherapy in resectable NSCLC. In the present study, unfavorable post-treatment nodal status or poor nodal response was associated with poorer OS and DFS across clinically comparable but heterogeneous nodal-response frameworks. However, because the available evidence is limited to a small number of retrospective studies with heterogeneous subgroup definitions and outcome reporting, ypN status should be regarded as a promising rather than validated postoperative prognostic factor. Future prospective multicenter studies using standardized pathological classifications and uniform survival endpoints are needed to clarify how ypN status should be integrated with primary-tumor response, pretreatment stage, and other postoperative risk markers.

## Data Availability

The data analyzed in this study is subject to the following licenses/restrictions: All data generated or analyzed during this study are included in this published article and its **Supplementary Material Files**. Additional details are available from the corresponding author upon reasonable request. Requests to access these datasets should be directed to Xuehongsheng1235@163.com.

## Supplementary material

Supplementary Figure 1 Sensitivity analysis of overall survival (OS) after replacing the prespecified comparison from Guo (10) with cN1→ypN+ versus cN1→ypN−, presented as a fixed-effect forest plot. OS, overall survival; HR, hazard ratio; CI, confidence interval.

Supplementary Figure 2 Sensitivity analysis of overall survival (OS) after replacing the prespecified comparison from Guo (10) with cN2→ypN− versus cN1→ypN−, presented as a fixed-effect forest plot. OS, overall survival; HR, hazard ratio; CI, confidence interval.

Supplementary Figure 3 Sensitivity analysis of overall survival (OS) after replacing the prespecified comparison from Ma (11) with downstaged N0 versus natural N0, presented as a random-effects forest plot. OS, overall survival; HR, hazard ratio; CI, confidence interval.

Supplementary Figure 4 Exploratory pooled analysis of overall survival (OS) including all five eligible comparisons reported by Guo (10) and Ma (11), presented as a fixed-effect forest plot. This analysis should be interpreted cautiously because some comparisons originated from the same study and shared a common reference group. OS, overall survival; HR, hazard ratio; CI, confidence interval.

Supplementary Figure 5 Sensitivity analysis of disease-free survival (DFS) after replacing the prespecified comparison from Deng (9) with ypN1-N2 versus ypN0, presented as a fixed-effect forest plot. DFS, disease-free survival; HR, hazard ratio; CI, confidence interval.

Supplementary Figure 6 Sensitivity analysis of disease-free survival (DFS) after replacing the prespecified comparison from Ma (11) with downstaged N0 versus natural N0, presented as a fixed-effect forest plot. DFS, disease-free survival; HR, hazard ratio; CI, confidence interval.

Supplementary Figure 7 Exploratory pooled analysis of disease-free survival (DFS) including all four eligible comparisons reported by Deng (9) and Ma (11), presented as a fixed-effect forest plot. This analysis should be interpreted cautiously because some comparisons originated from the same study and were not statistically independent. DFS, disease-free survival; HR, hazard ratio; CI, confidence interval.

Supplementary Figure 8 Single-study forest plot showing the recurrence-free survival (RFS) associations reported by Pan (13), using MPR ypN0 as the reference group. Because all displayed comparisons originated from the same study and shared a common reference group, no pooled summary estimate, pooled diamond, heterogeneity statistic, or overall-effect test is shown. RFS, recurrence-free survival; MPR, major pathological response; HR, hazard ratio; CI, confidence interval. Pan (13) = non-MPR ypN0 versus MPR ypN0; Pan (13)* = MPR ypN+ versus MPR ypN0; Pan (13)** = non-MPR ypN+ versus MPR ypN0.
