# Supplementary figures and images for: Prognostic significance of ypN status after neoadjuvant chemoimmunotherapy in resectable NSCLC: a systematic review and meta-analysis
Source: Front Oncol. 2026 May 22;16:1842157. doi: 10.3389/fonc.2026.1842157 (PMC13236610; doi:10.3389/fonc.2026.1842157)

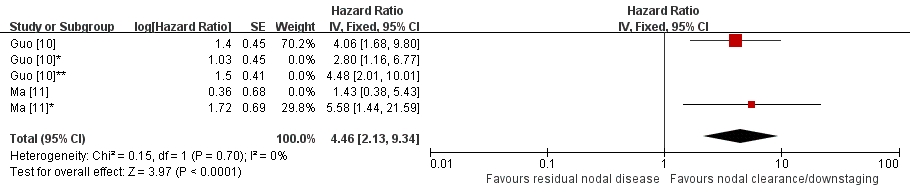

Supplement: Supplementary Figure 1 — Sensitivity analysis of overall survival (OS) after replacing the prespecified comparison from Guo (10) with cN1→ypN+ versus cN1→ypN−, presented as a fixed-effect forest plot. OS, overall survival; HR, hazard ratio; CI, confidence interval. [file Image1.jpeg]

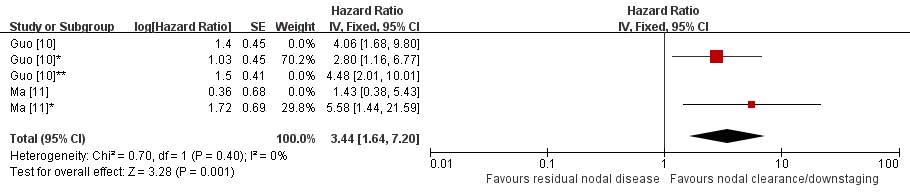

Supplement: Supplementary Figure 2 — Sensitivity analysis of overall survival (OS) after replacing the prespecified comparison from Guo (10) with cN2→ypN− versus cN1→ypN−, presented as a fixed-effect forest plot. OS, overall survival; HR, hazard ratio; CI, confidence interval. [file Image2.jpeg]

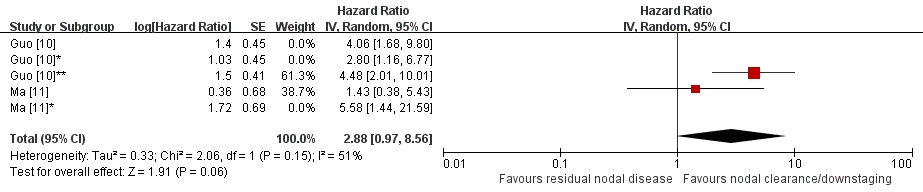

Supplement: Supplementary Figure 3 — Sensitivity analysis of overall survival (OS) after replacing the prespecified comparison from Ma (11) with downstaged N0 versus natural N0, presented as a random-effects forest plot. OS, overall survival; HR, hazard ratio; CI, confidence interval. [file Image3.jpeg]

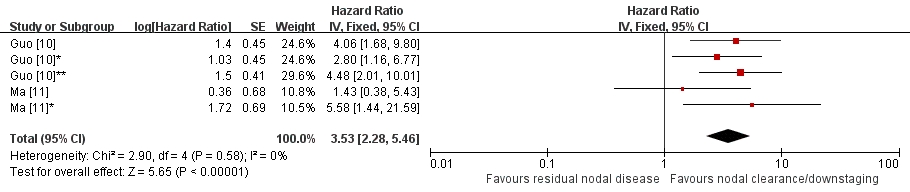

Supplement: Supplementary Figure 4 — Exploratory pooled analysis of overall survival (OS) including all five eligible comparisons reported by Guo (10) and Ma (11), presented as a fixed-effect forest plot. This analysis should be interpreted cautiously because some comparisons originated from the same study and shared a common reference group. OS, overall survival; HR, hazard ratio; CI, confidence interval. [file Image4.jpeg]

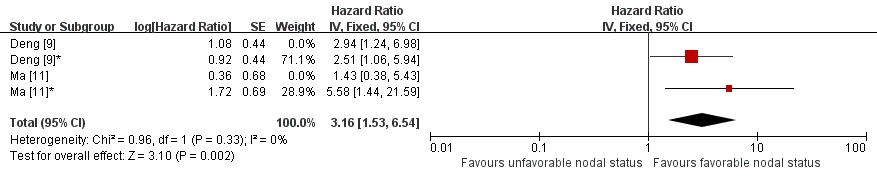

Supplement: Supplementary Figure 5 — Sensitivity analysis of disease-free survival (DFS) after replacing the prespecified comparison from Deng (9) with ypN1-N2 versus ypN0, presented as a fixed-effect forest plot. DFS, disease-free survival; HR, hazard ratio; CI, confidence interval. [file Image5.jpeg]

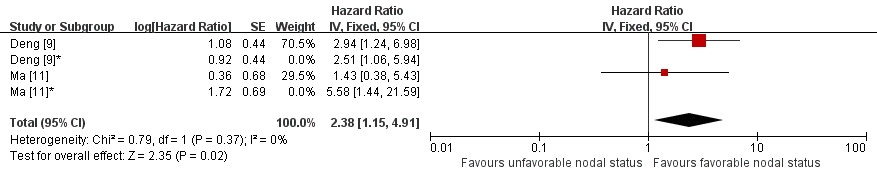

Supplement: Supplementary Figure 6 — Sensitivity analysis of disease-free survival (DFS) after replacing the prespecified comparison from Ma (11) with downstaged N0 versus natural N0, presented as a fixed-effect forest plot. DFS, disease-free survival; HR, hazard ratio; CI, confidence interval. [file Image6.jpeg]

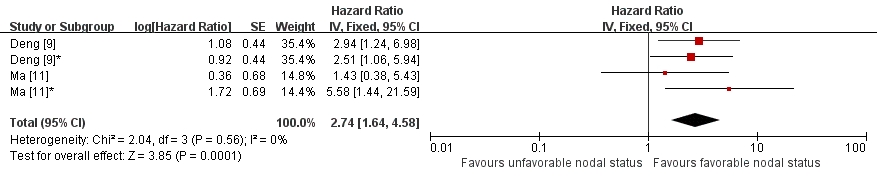

Supplement: Supplementary Figure 7 — Exploratory pooled analysis of disease-free survival (DFS) including all four eligible comparisons reported by Deng (9) and Ma (11), presented as a fixed-effect forest plot. This analysis should be interpreted cautiously because some comparisons originated from the same study and were not statistically independent. DFS, disease-free survival; HR, hazard ratio; CI, confidence interval. [file Image7.jpeg]

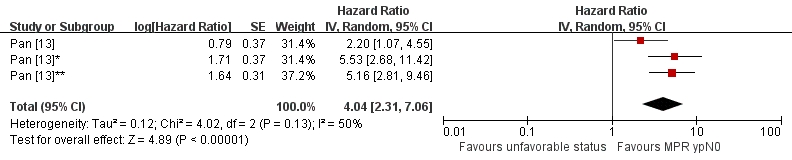

Supplement: Supplementary Figure 8 — Single-study forest plot showing the recurrence-free survival (RFS) associations reported by Pan (13), using MPR ypN0 as the reference group. Because all displayed comparisons originated from the same study and shared a common reference group, no pooled summary estimate, pooled diamond, heterogeneity statistic, or overall-effect test is shown. RFS, recurrence-free survival; MPR, major pathological response; HR, hazard ratio; CI, confidence interval. Pan (13) = non-MPR ypN0 versus MPR ypN0; Pan (13)* = MPR ypN+ versus MPR ypN0; Pan (13)** = non-MPR ypN+ versus MPR ypN0. [file Image8.jpeg]
