# Supplementary material for: Prognostic significance of ypN status after neoadjuvant chemoimmunotherapy in resectable NSCLC: a systematic review and meta-analysis
Source: Front Oncol. 2026 May 22;16:1842157. doi: 10.3389/fonc.2026.1842157 (PMC13236610; doi:10.3389/fonc.2026.1842157)
Supplement: Supplementary file 9 [file Table1.docx]

**Supplementary Methods: Literature search strategy**

We systematically searched PubMed, Embase, Web of Science Core Collection, Scopus, and the Cochrane Library to identify studies evaluating the prognostic significance of ypN status after neoadjuvant chemoimmunotherapy in resectable non-small cell lung cancer (NSCLC). The searches were performed from database inception to 26 February 2026. In addition, the reference lists of all included studies and relevant reviews were manually screened to identify any further eligible studies. The detailed search strategies for each database are provided below.

**1. PubMed**

**Database:** PubMed (MEDLINE)

**Date range:** from database inception to 26 February 2026

**Search field:** Title/Abstract and related indexing terms

**Search strategy:**

((("Carcinoma, Non-Small-Cell Lung"[Mesh]) OR "non-small cell lung cancer" OR "non-small-cell lung cancer" OR NSCLC OR "lung adenocarcinoma" OR "lung squamous cell carcinoma" OR "resectable NSCLC" OR "resectable lung cancer" OR "lung neoplasm")
AND
("neoadjuvant chemoimmunotherapy" OR "neoadjuvant immunochemotherapy" OR "neoadjuvant immunotherapy plus chemotherapy" OR "neoadjuvant chemotherapy plus immunotherapy" OR "preoperative chemoimmunotherapy" OR "preoperative immunochemotherapy" OR "neoadjuvant immuno-chemotherapy" OR "neoadjuvant chemo-immunotherapy" OR "neoadjuvant PD-1" OR "neoadjuvant PD-L1" OR "neoadjuvant immune checkpoint inhibitor" OR "neoadjuvant checkpoint inhibitor" OR nivolumab OR pembrolizumab OR atezolizumab OR durvalumab OR sintilimab OR camrelizumab OR toripalimab OR tislelizumab OR cemiplimab OR "nivolumab plus chemotherapy" OR "pembrolizumab plus chemotherapy" OR "atezolizumab plus chemotherapy" OR "durvalumab plus chemotherapy" OR "sintilimab plus chemotherapy" OR "camrelizumab plus chemotherapy" OR "toripalimab plus chemotherapy" OR "tislelizumab plus chemotherapy")
AND
("ypN" OR "ypN status" OR ypN0 OR "pathologic nodal status" OR "pathological nodal status" OR "postoperative nodal status" OR "residual nodal disease" OR "nodal status" OR "nodal downstaging" OR "lymph node downstaging" OR "lymph node status" OR "lymph node response" OR "nodal response")
AND
(prognos* OR survival OR outcome* OR "overall survival" OR "disease-free survival" OR DFS OR "event-free survival" OR EFS OR "recurrence-free survival" OR RFS OR "progression-free survival" OR PFS OR recurren*))
NOT
(review[Publication Type] OR systematicreview OR "systematic review" OR meta-analysis OR metaanalysis OR editorial[Publication Type] OR comment[Publication Type] OR letter[Publication Type] OR case reports[Publication Type] OR "case report" OR "case reports" OR "conference abstract" OR "meeting abstract" OR "oral presentation" OR poster OR "poster presentation"))

**2. Embase**

**Database:** Embase

**Date range:** from database inception to 26 February 2026

**Search strategy:**

('non-small cell lung cancer' OR 'non-small-cell lung cancer' OR NSCLC OR 'lung adenocarcinoma' OR 'lung squamous cell carcinoma' OR 'resectable NSCLC' OR 'resectable lung cancer' OR 'lung neoplasm')
AND
('neoadjuvant chemoimmunotherapy' OR 'neoadjuvant immunochemotherapy' OR 'neoadjuvant immunotherapy plus chemotherapy' OR 'neoadjuvant chemotherapy plus immunotherapy' OR 'preoperative chemoimmunotherapy' OR 'preoperative immunochemotherapy' OR 'neoadjuvant immuno-chemotherapy' OR 'neoadjuvant chemo-immunotherapy' OR 'neoadjuvant PD-1' OR 'neoadjuvant PD-L1' OR 'neoadjuvant immune checkpoint inhibitor' OR 'neoadjuvant checkpoint inhibitor' OR nivolumab OR pembrolizumab OR atezolizumab OR durvalumab OR sintilimab OR camrelizumab OR toripalimab OR tislelizumab OR cemiplimab OR 'nivolumab plus chemotherapy' OR 'pembrolizumab plus chemotherapy' OR 'atezolizumab plus chemotherapy' OR 'durvalumab plus chemotherapy' OR 'sintilimab plus chemotherapy' OR 'camrelizumab plus chemotherapy' OR 'toripalimab plus chemotherapy' OR 'tislelizumab plus chemotherapy')
AND
('ypN' OR 'ypN status' OR ypN0 OR 'pathologic nodal status' OR 'pathological nodal status' OR 'postoperative nodal status' OR 'residual nodal disease' OR 'nodal status' OR 'nodal downstaging' OR 'lymph node downstaging' OR 'lymph node status' OR 'lymph node response' OR 'nodal response')
AND
(prognos* OR survival OR outcome* OR 'overall survival' OR 'disease-free survival' OR DFS OR 'event-free survival' OR EFS OR 'recurrence-free survival' OR RFS OR 'progression-free survival' OR PFS OR recurren*)
NOT
('review' OR 'systematic review' OR 'meta analysis' OR 'editorial' OR 'letter' OR 'note' OR 'case report' OR 'conference abstract' OR 'conference paper' OR 'conference review' OR 'conference proceeding' OR poster OR 'oral presentation')

**3. Web of Science Core Collection**

**Database:** Web of Science Core Collection

**Date range:** from database inception to 26 February 2026

**Search field:** Topic (TS)

**Search strategy:**

TS=(("non-small cell lung cancer" OR "non-small-cell lung cancer" OR NSCLC OR "lung adenocarcinoma" OR "lung squamous cell carcinoma" OR "resectable NSCLC" OR "resectable lung cancer" OR "lung neoplasm")
AND
("neoadjuvant chemoimmunotherapy" OR "neoadjuvant immunochemotherapy" OR "neoadjuvant immunotherapy plus chemotherapy" OR "neoadjuvant chemotherapy plus immunotherapy" OR "preoperative chemoimmunotherapy" OR "preoperative immunochemotherapy" OR "neoadjuvant immuno-chemotherapy" OR "neoadjuvant chemo-immunotherapy" OR "neoadjuvant PD-1" OR "neoadjuvant PD-L1" OR "neoadjuvant immune checkpoint inhibitor" OR "neoadjuvant checkpoint inhibitor"
OR nivolumab OR pembrolizumab OR atezolizumab OR durvalumab OR sintilimab OR camrelizumab OR toripalimab OR tislelizumab OR cemiplimab OR "nivolumab plus chemotherapy" OR "pembrolizumab plus chemotherapy" OR "atezolizumab plus chemotherapy" OR "durvalumab plus chemotherapy" OR "sintilimab plus chemotherapy" OR "camrelizumab plus chemotherapy" OR "toripalimab plus chemotherapy" OR "tislelizumab plus chemotherapy")
AND
("ypN" OR "ypN status" OR ypN0 OR "pathologic nodal status" OR "pathological nodal status" OR "postoperative nodal status" OR "residual nodal disease" OR "nodal status" OR "nodal downstaging" OR "lymph node downstaging" OR "lymph node status" OR "lymph node response" OR "nodal response")
AND
(prognos* OR survival OR outcome* OR "overall survival" OR "disease-free survival" OR DFS OR "event-free survival" OR EFS OR "recurrence-free survival" OR RFS OR "progression-free survival" OR PFS OR recurren*))
NOT TS=("systematic review" OR "meta-analysis" OR metaanalysis OR review OR editorial OR letter OR "case report" OR "case reports" OR "conference abstract" OR "meeting abstract" OR poster OR "oral presentation")

**4. Scopus**

**Database:** Scopus

**Date range:** from database inception to 26 February 2026

**Search field:** TITLE-ABS-KEY

**Search strategy:**

TITLE-ABS-KEY((("non-small cell lung cancer" OR "non-small-cell lung cancer" OR NSCLC OR "lung adenocarcinoma" OR "lung squamous cell carcinoma" OR "resectable NSCLC" OR "resectable lung cancer" OR "lung neoplasm")
AND
("neoadjuvant chemoimmunotherapy" OR "neoadjuvant immunochemotherapy" OR "neoadjuvant immunotherapy plus chemotherapy" OR "neoadjuvant chemotherapy plus immunotherapy" OR "preoperative chemoimmunotherapy" OR "preoperative immunochemotherapy" OR "neoadjuvant immuno-chemotherapy" OR "neoadjuvant chemo-immunotherapy" OR "neoadjuvant PD-1" OR "neoadjuvant PD-L1" OR "neoadjuvant immune checkpoint inhibitor" OR "neoadjuvant checkpoint inhibitor"
OR nivolumab OR pembrolizumab OR atezolizumab OR durvalumab OR sintilimab OR camrelizumab OR toripalimab OR tislelizumab OR cemiplimab OR "nivolumab plus chemotherapy" OR "pembrolizumab plus chemotherapy" OR "atezolizumab plus chemotherapy" OR "durvalumab plus chemotherapy" OR "sintilimab plus chemotherapy" OR "camrelizumab plus chemotherapy" OR "toripalimab plus chemotherapy" OR "tislelizumab plus chemotherapy")
AND
("ypN" OR "ypN status" OR ypN0 OR "pathologic nodal status" OR "pathological nodal status" OR "postoperative nodal status" OR "residual nodal disease" OR "nodal status" OR "nodal downstaging" OR "lymph node downstaging" OR "lymph node status" OR "lymph node response" OR "nodal response")
AND
(prognos* OR survival OR outcome* OR "overall survival" OR "disease-free survival" OR DFS OR "event-free survival" OR EFS OR "recurrence-free survival" OR RFS OR "progression-free survival" OR PFS OR recurren*)))
AND NOT TITLE-ABS-KEY("systematic review" OR "meta-analysis" OR metaanalysis OR review OR editorial OR letter OR "case report" OR "case reports" OR "conference abstract" OR "meeting abstract" OR poster OR "oral presentation")

**5. Cochrane Library**

**Database:** Cochrane Library

**Date range:** from database inception to 26 February 2026

**Search strategy:**

(("non-small cell lung cancer" OR "non-small-cell lung cancer" OR NSCLC OR "lung adenocarcinoma" OR "lung squamous cell carcinoma" OR "resectable NSCLC" OR "resectable lung cancer" OR "lung neoplasm")
AND
("neoadjuvant chemoimmunotherapy" OR "neoadjuvant immunochemotherapy" OR "neoadjuvant immunotherapy plus chemotherapy" OR "neoadjuvant chemotherapy plus immunotherapy" OR "preoperative chemoimmunotherapy" OR "preoperative immunochemotherapy" OR "neoadjuvant immuno-chemotherapy" OR "neoadjuvant chemo-immunotherapy" OR "neoadjuvant PD-1" OR "neoadjuvant PD-L1" OR "neoadjuvant immune checkpoint inhibitor" OR "neoadjuvant checkpoint inhibitor" OR nivolumab OR pembrolizumab OR atezolizumab OR durvalumab OR sintilimab OR camrelizumab OR toripalimab OR tislelizumab OR cemiplimab OR "nivolumab plus chemotherapy" OR "pembrolizumab plus chemotherapy" OR "atezolizumab plus chemotherapy" OR "durvalumab plus chemotherapy" OR "sintilimab plus chemotherapy" OR "camrelizumab plus chemotherapy" OR "toripalimab plus chemotherapy" OR "tislelizumab plus chemotherapy")
AND
("ypN" OR "ypN status" OR ypN0 OR "pathologic nodal status" OR "pathological nodal status" OR "postoperative nodal status" OR "residual nodal disease" OR "nodal status" OR "nodal downstaging" OR "lymph node downstaging" OR "lymph node status" OR "lymph node response" OR "nodal response")
AND
(prognos* OR survival OR outcome* OR "overall survival" OR "disease-free survival" OR DFS OR "event-free survival" OR EFS OR "recurrence-free survival" OR RFS OR "progression-free survival" OR PFS OR recurren*))
NOT
("systematic review" OR "meta-analysis" OR metaanalysis OR review OR editorial OR letter OR "case report" OR "case reports" OR "conference abstract" OR "meeting abstract" OR poster OR "oral presentation")
