# Supplementary material for: Prognostic significance of ypN status after neoadjuvant chemoimmunotherapy in resectable NSCLC: a systematic review and meta-analysis
Source: Front Oncol. 2026 May 22;16:1842157. doi: 10.3389/fonc.2026.1842157 (PMC13236610; doi:10.3389/fonc.2026.1842157)
Supplement: Supplementary file 10 [file Table2.docx]

| Supplementary Table S1. Detailed extraction and transformation of time-to-event outcomes according to post-neoadjuvant nodal status in the included studies | | | | | | | | | | | | | | | | | | | | | | | | | | | | | | | | | | | | | | | | | |
| --- | --- | --- | --- | --- | --- | --- | --- | --- | --- | --- | --- | --- | --- | --- | --- | --- | --- | --- | --- | --- | --- | --- | --- | --- | --- | --- | --- | --- | --- | --- | --- | --- | --- | --- | --- | --- | --- | --- | --- | --- | --- |
| Authors | Year | Country | Design | Population / disease entity | Total (n) | Male (n) | Female (n) | Smoking hx | Histology overall | Histology by subgroup | Age overall | Stage range | cT stage | cN stage | ypT stage | ypN0 (n) | ypN+ (n) | Other nodal grouping reported | Nodal classification framework | Tx modality | Adjuvant Tx | Main clinicopathological variables reported | MPR | pCR | LN metastasis / nodal burden | Recurrence pattern | Survival outcomes reported | Exact survival group labels | OS HR | OS 95% CI | DFS HR | DFS 95% CI | PFS HR | PFS 95% CI | RFS HR | RFS 95% CI | Response data | MVA | Adjusted variables | HR extraction type | Study period |
| Deng et al.[9] | 2023 | China | Retrospective cohort | Initial cStage III NSCLC treated with neoadjuvant immunochemotherapy and radical-intent surgery | 53 | 47 | 6 | Reported | Squamous cell carcinoma 35/53; non-squamous histology 18/53 | NR | NR | cStage III | Reported | All patients had paired involved LNs reviewed | Reported | 28 | 25 | ypN0 versus ypN1-N2; mLN-MPR also evaluated | Primary tumor and paired metastatic LNs pathologically reviewed; LN response assessed by mLN-MPR and ypN stage | Neoadjuvant immunochemotherapy + surgery | Adjuvant treatment reported; 40/53 received adjuvant treatment | Primary tumor pathological response; paired metastatic LN pathological response; RVT in LNs; pathological nodal stage | 31/53 (58.5%) | NR | All patients had initial LN metastasis disease and paired involved LNs reviewed | NR | DFS | ypN0 versus ypN1-N2 | NR | NR | 0.4 | 0.17-0.94 | NR | NR | NR | NR | mLN-MPR was present in 34/53 (64.2%); PT-MPR was present in 31/53 (58.5%); ypN0 was achieved in 28/53 (52.8%); mLN-MPR(+) showed HR 0.34 (95% CI 0.14-0.78), and ypN0 showed HR 0.40 (95% CI 0.17-0.94) for DFS | No | Not applicable | Directly reported | 2020 to 2021 |
| Du et al. [12] | 2023 | China | Retrospective cohort | Resectable NSCLC with baseline cN1 or cN2 nodal disease treated with neoadjuvant chemoimmunotherapy and surgery | 75 | 69 | 6 | Current/former smokers 67; never smokers 8 | Squamous cell carcinoma 60/75; adenocarcinoma 15/75 | NR | 64 years (range 41-78) | Stage IIb-IIIb | Reported | cN1: 26; cN2: 49 | Reported | 54 | 21 | Nodal clearance versus nodal residual disease | Post-treatment nodal status assessed as nodal clearance versus nodal residual disease | Neoadjuvant chemoimmunotherapy + surgery | 50 patients received at least 1 cycle of adjuvant chemotherapy, immunotherapy, or both | Age; sex; smoking history; histology; baseline nodal burden; MPR; pCR; TRAEs | 45/75 (60%) | 27/75 (36%) | Multiple nodal metastasis reported | NR | PFS; OS | Nodal clearance group versus nodal residual group | NR | NR | NR | NR | NR | NR | NR | NR | Nodal clearance in 34/49 (67%) of cN2 and 20/26 (77%) of cN1 patients; 12- and 18-month PFS were 90.1% and 83.6% in the nodal clearance group versus 70.1% and 63.7% in the nodal residual group; 12- and 18-month OS were 100% and 96.6% versus 85.4% and 78.8%, respectively | No | Not applicable | Not directly extractable; Kaplan-Meier/log-rank only | January 2021 to June 2022 |
| Guo et al. [10] | 2025 | United States | Retrospective database study | Resectable NSCLC with baseline cN1 or cN2 disease treated with neoadjuvant chemoimmunotherapy and surgery | 621 | NR | NR | NR | NR | NR | NR | NR | Reported | cN1: 229; cN2: 392 | Reported | 293 | 328 | cN1 to ypN0: 135; cN1 to ypN+: 94; cN2 to ypN0: 158; cN2 to ypN+: 234; overall ypN0: 293; overall ypN1: 108; overall ypN2: 220 | NCDB analysis stratified by baseline cN stage and post-treatment ypN stage; ypN- defined as ypN0 and ypN+ defined as ypN1 or ypN2 | Neoadjuvant chemoimmunotherapy + surgery | Adjuvant chemoimmunotherapy and adjuvant radiation reported | Age at diagnosis; sex; race; facility type; insurance status; Charlson-Deyo comorbidity index; histology; tumor size; cT stage; cN stage; time to systemic therapy; time to immunotherapy; time to surgery; operation; surgical approach; postoperative length of stay; 30-day and 90-day mortality; surgical margins; regional LNs examined; regional LNs positive; ypT stage; adjuvant chemoimmunotherapy; adjuvant radiation | NR | NR | Baseline cN1 or cN2 only; regional nodes examined and regional nodes positive reported | NR | OS | cN1 to ypN0 versus cN1 to ypN+ versus cN2 to ypN0 versus cN2 to ypN+ | 4.49 | 1.99-10.1 | NR | NR | NR | NR | NR | NR | Five-year OS was 76% for cN1 and 63% for cN2. Five-year OS by post-therapy nodal stage was 84% for ypN0, 64% for ypN1, and 51% for ypN2. Among cN1 patients, 5-year OS was 91% for ypN0 versus 57% for ypN+; among cN2 patients, 79% for ypN0 versus 54% for ypN+ | Yes | Multivariable model adjusted for facility type, surgical approach, surgical margin status, number of regional LNs examined, ypT stage, and adjuvant chemoimmunotherapy | Directly reported (multivariable HRs for combined cN/ypN groups, not simple overall ypN0 versus ypN+) | 2017 to 2021 |
| Ma et al. [11] | 2025 | China | Retrospective multicenter cohort | NSCLC after neoadjuvant chemoimmunotherapy and surgery | 186 | 167 | 19 | Current/former smokers 121; never smokers 65 | Adenocarcinoma 42/186; squamous cell carcinoma 144/186 | Natural N0 group: adenocarcinoma 5/34, squamous cell carcinoma 29/34; downstaged N0 group: adenocarcinoma 21/95, squamous cell carcinoma 74/95; ypN+ group: adenocarcinoma 16/57, squamous cell carcinoma 41/57 | NR | Stage IB-IIIB | cT1 10; cT2 90; cT3 55; cT4 31 | cN0 35; cN1 30; cN2 121 | ypT0 74; ypT1 71; ypT2 31; ypT3 8; ypT4 2 | 129 | 57 | Natural node-negative (cN0/ypN0): 34; downstaged node-negative (cN+/ypN0): 95; ypN+ (cN+/ypN+): 57 | LN status grouped as cN0/ypN0, cN+/ypN0, and cN+/ypN+ | Neoadjuvant chemoimmunotherapy + surgery | NR | Pretreatment LN status; cT stage; cN stage; ypT stage; ypN stage; MPR; number of involved LNs; number of involved N stations; recurrence pattern | 116/186 (62.4%) | 74/186 (39.8%) | Clinical N0 35; clinical N1 30; clinical N2 121; number of involved LNs <=3 in 168/186 and >3 in 18/186; number of involved N stations <2 in 156/186 and >=2 in 30/186 | Local recurrence 15/186; distant recurrence 19/186 | DFS; OS | cN0/ypN0 versus cN+/ypN0 versus cN+/ypN+ | 11.473 | 1.237-106.379 | 5.568 | 1.435-21.605 | NR | NR | NR | NR | Natural N0: 1-year and 2-year DFS 100.0% and 93.8%; downstaged N0: 95.8% and 91.3%; ypN+: 80.7% and 62.3%. Natural N0: 1-year and 2-year OS 100.0% and 96.0%; downstaged N0: 98.9% and 97.8%; ypN+: 94.7% and 73.8%. No significant DFS or OS difference between natural N0 and downstaged N0; both had significantly better outcomes than ypN+ | Yes | Multivariable model included ypT stage, MPR, number of involved LNs, number of involved N stations, and LN status; clinical N stage and pathological N stage were excluded from multivariable analysis because of high correlation with LN status | Directly reported (multivariable HRs for LN status) | 2019 to 2022 |
| Pan et al. [13] | 2026 | China | Retrospective multicenter cohort | cStage IB-III resectable NSCLC treated with neoadjuvant chemoimmunotherapy followed by curative-intent surgery | 363 | 330 | 33 | Never smoker 105; smoker or ex-smoker 258 | LUAD 77/363; LUSC 263/363; others 23/363 | MPR ypN0 group: LUAD 26/195, LUSC 153/195, others 16/195; non-MPR ypN0 group: LUAD 16/65, LUSC 47/65, others 2/65; MPR ypN+ group: LUAD 3/31, LUSC 27/31, others 1/31; non-MPR ypN+ group: LUAD 32/72, LUSC 36/72, others 4/72 | 65.00 years [IQR 60.00-70.00] | cStage IB-III | cT1 56; cT2 165; cT3 87; cT4 55 | cN0 82; cN1 85; cN2 196 | ypT0 139; ypT1 188; ypT2 24; ypT3 7; ypT4 5 | 260 | 103 | MPR ypN0: 195; non-MPR ypN0: 65; MPR ypN+: 31; non-MPR ypN+: 72 | Post-treatment ypN dichotomized as ypN0 versus ypN+ and integrated with MPR into four prognostic groups | Neoadjuvant chemoimmunotherapy + surgery | Adjuvant IO yes 134/363 and no 229/363; by subgroup: MPR ypN0 64/195, non-MPR ypN0 24/65, MPR ypN+ 13/31, non-MPR ypN+ 33/72 | Age; sex; smoking history; BMI; FEV1; TLCO; ECOG PS; tumor location; tumor length; cT stage; cN stage; clinical stage; PD-L1; neoadjuvant cycles; surgery methods; histological type; ypT stage; ypN stage; pathological stage; MPR; pCR; adjuvant IO | 226/363 (62.3%) | 129/363 (35.5%) | ypN0 260/363; ypN1 56/363; ypN2 47/363 | Reported; non-MPR ypN+ group had the highest recurrence burden with increased LR and DM | RFS; OS | ypN0 versus ypN+; also MPR ypN0 versus non-MPR ypN0 versus MPR ypN+ versus non-MPR ypN+ | NR | NR | NR | NR | NR | NR | NR | NR | Median follow-up 27.8 months (95% CI 26.5-29.2); 2-year RFS 77.7% and 2-year OS 92.9% in the overall cohort. ypN0 showed significantly better RFS (P < .001) and OS (P = .006) than ypN+. Two-year RFS by group: MPR ypN0 90.7%, non-MPR ypN0 75.2%, MPR ypN+ 62.4%, non-MPR ypN+ 55.2%. Two-year OS by group: MPR ypN0 93.5%, non-MPR ypN0 96.7%, MPR ypN+ 93.1%, non-MPR ypN+ 87.9% | Yes | Multivariable model included variables with P < 0.05 in univariable analysis plus adjuvant immunotherapy; with MPR ypN0 as reference, non-MPR ypN0 HR 2.21 (95% CI 1.07-4.56), MPR ypN+ HR 5.54 (95% CI 2.66-11.54), and non-MPR ypN+ HR 5.18 (95% CI 2.80-9.55) for RFS | Not directly extractable for simple ypN0 versus ypN+; directly reported for combined MPR-ypN groups | January 2020 to December 2024 |
| NSCLC, non-small cell lung cancer; cStage, clinical stage; cT, clinical T stage; cN, clinical nodal stage; ypT, post-treatment pathological T stage; ypN, post-treatment pathological nodal stage; ypN0, post-treatment pathological node-negative status; ypN+, post-treatment pathological node-positive status; LN, lymph node; LNs, lymph nodes; Tx, treatment; MPR, major pathological response; mLN-MPR, metastatic lymph node major pathological response; pCR, pathological complete response; RVT, residual viable tumor; PFS, progression-free survival; DFS, disease-free survival; RFS, recurrence-free survival; OS, overall survival; HR, hazard ratio; CI, confidence interval; MVA, multivariable analysis; IO, immunotherapy; NCDB, National Cancer Database; TRAEs, treatment-related adverse events; hx, history; LUAD, lung adenocarcinoma; LUSC, lung squamous cell carcinoma; BMI, body mass index; FEV1, forced expiratory volume in 1 second; TLCO, transfer factor of the lung for carbon monoxide; ECOG PS, Eastern Cooperative Oncology Group performance status; PD-L1, programmed death-ligand 1; LR, local recurrence; DM, distant metastasis; IQR, interquartile range; NR, not reported. Reported but not directly extractable indicates that the relevant result was described in the original article, but a numerical effect estimate suitable for direct quantitative synthesis was not available from the published text, tables, or figures. | | | | | | | | | | | | | | | | | | | | | | | | | | | | | | | | | | | | | | | | | |
